# Associations of Maternal Milk Feeding With Neurodevelopmental Outcomes at 7 Years of Age in Former Preterm Infants

**DOI:** 10.1001/jamanetworkopen.2022.21608

**Published:** 2022-07-13

**Authors:** Mandy B. Belfort, Emma Knight, Shikha Chandarana, Emmanuella Ikem, Jacqueline F. Gould, Carmel T. Collins, Maria Makrides, Robert A. Gibson, Peter J. Anderson, Karen Simmer, Henning Tiemeier, Alice Rumbold

**Affiliations:** 1 Department of Pediatric Newborn Medicine, Brigham and Women’s Hospital, Boston, Massachusetts; 2 South Australian Health and Medical Research Institute Women and Kids, Adelaide, Australia; 3 University of Adelaide School of Public Health, Adelaide, Australia; 4 Department of Social and Behavioral Sciences, Harvard T.H. Chan School of Public Health, Boston, Massachusetts; 5 Department of Prevention and Community Health, Milken Institute of Public Health, George Washington University, Washington, DC; 6 Department of Pediatrics, The University of Melbourne, Royal Children’s Hospital, Victoria, Australia; 7 Murdoch Children’s Research Institute, Melbourne, Victoria, Australia; 8 Centre for Neonatal Research and Education, School of Pediatrics and Child Health, University of Western Australia, Perth, Australia

## Abstract

**Importance:**

Maternal milk feeding may have unique long-term neurodevelopmental benefits in very preterm infants.

**Objective:**

To examine the extent to which maternal milk feeding after very preterm birth is associated with cognitive, academic, and behavioral outcomes at school age.

**Design, Setting, and Participants:**

This prospective cohort study assessed 586 infants born at less than 33 weeks’ gestation at 5 Australian perinatal centers and enrolled in the Docosahexaenoic Acid for Improvement of Neurodevelopmental Outcomes study (January 1, 2001, to December 31, 2005) who were evaluated at a corrected age of 7 years. The statistical analysis was completed on January 19, 2022.

**Exposures:**

Maternal milk intake, including mean volume (milliliters per kilogram per day) during neonatal hospitalization and total duration (in months).

**Main Outcomes and Measures:**

Neurodevelopmental outcomes at 7 years of age were (1) IQ (Wechsler Abbreviated Scale of Intelligence), (2) academic achievement (Wide Range Achievement Test, Fourth Edition), (3) symptoms of attention-deficit/hyperactivity disorder (ADHD) (Conners Third Edition ADHD Index, parent reported), (4) executive function (Behavior Rating Inventory of Executive Functioning, parent reported), and (5) behavior (Strengths and Difficulties Questionnaire, parent reported).

**Results:**

A total of 586 infants (mean [SD] gestational age at birth, 29.6 [2.3] weeks; 314 male [53.6%]) born to 486 mothers (mean [SD] age, 30.6 [5.5] years; 447 [92.0%] White) were included. Mean (SD) maternal milk intake in the neonatal intensive care unit was 99 (48) mL/kg daily, and mean (SD) maternal milk duration was 5.1 (5.4) months. Mean (SD) full-scale IQ was 98.5 (13.3) points. After covariate adjustment, higher maternal milk intake during the neonatal hospitalization was associated with higher performance IQ (0.67 points per additional 25 mL/kg daily; 95% CI, 0.10-1.23 points), reading scores (1.14 points per 25 mL/kg daily; 95% CI, 0.39-1.89 points), and math scores (0.76 points per 25 mL/kg daily; 95% CI, 0.14-1.37 points) and fewer ADHD symptoms (−1.08 points per 25 mL/kg daily; 95% CI, −1.96 to −0.20 points). Longer duration of maternal milk intake was associated with higher reading (0.33 points per additional month; 95% CI, 0.03-0.63 points), spelling (0.31 points per month; 95% CI, 0.01-0.62 points), and math (0.30 points per month; 95% CI, 0.03-0.58 points) scores. Maternal milk was not associated with improved full-scale IQ, verbal IQ, executive function, or behavior. Most associations were stronger among infants born at lower gestational ages, particularly less than 30 weeks (interaction *P* values <.01).

**Conclusions and Relevance:**

In this cohort study of preterm infants, maternal milk feeding during the neonatal hospitalization and after discharge were associated with better school-age performance IQ and academic achievement and with a reduction in ADHD symptoms, particularly among infants born at less than 30 weeks’ gestation.

## Key Points

**Question:**

What are the associations between maternal milk feeding and cognitive, academic, and behavioral outcomes of preterm infants born at less than 33 weeks’ gestation?

**Findings:**

In this cohort study of 586 preterm infants, higher maternal milk intake during neonatal hospitalization was associated with higher performance IQ, better academic achievement in reading and math, and fewer attention-deficit/hyperactivity symptoms at 7 years of age. These associations persisted after adjustment for clinical and social confounders and were generally more pronounced among infants born at lower gestational ages.

**Meaning:**

These findings affirm recommendations to provide maternal milk to hospitalized very preterm infants based on potential long-term benefits to neurodevelopment.

## Teaser

This cohort study examines whether maternal milk feeding after very preterm birth is associated with cognitive, academic, and behavioral outcomes at school age and whether associations differ according to gestational age at birth, fetal growth status, and infant sex.

## Introduction

Compelling evidence links more prolonged and exclusive breastfeeding in infancy with improved neurodevelopmental outcomes in childhood.^1,2^ Postulated mechanisms include nutrients and nonnutrient bioactive factors in maternal milk that benefit the infant brain during a critical period in development.^3^ Whether breastfeeding offers similar benefits to infants born very preterm (<32 weeks of gestation) is less certain. Findings from studies of full-term infants are not directly generalizable to the very preterm population given substantial differences in postnatal nutrient requirements^4^ and neurodevelopmental risk profiles.^5^ Furthermore, the very preterm infant is fed expressed maternal milk during the several-months-long period from preterm birth to term equivalent age, a period of susceptibility to brain injury^6^ and one that coincides with an earlier stage of brain development than when the full-term infant is fed maternal milk. It is plausible that maternal milk may have different effects at different stages of brain development. Furthermore, within cohorts of preterm infants, the beneficial effect of maternal milk may vary by gestational age and fetal growth status as well as by infant sex.

Some but not all previous studies^6,7^ have linked maternal milk feeding during neonatal hospitalization with better neurodevelopmental outcomes in childhood. Differences in brain structure at term-equivalent age associated with maternal milk feeding in the interval from birth to hospital discharge provide further evidence to support a beneficial effect.^8,9,10,11^ Most studies^6,7^ of maternal milk and neurodevelopment have focused on outcomes at 2 years or earlier, in part because of the challenge and expense of retaining large cohorts for many years. However, many important neurodevelopmental domains are not reliably assessable until school age. To date, few studies have investigated the association between maternal milk feeding during neonatal hospitalization and neurodevelopment at school age, and existing studies^7^ are limited by small sample size, which precludes adjustment for multiple confounders and stratified analyses, as well as by a lack of detailed data on the dose of maternal milk to which the infant was exposed. In addition, previous studies^6^ examined a limited range of neurodevelopmental outcomes. Our aims were to examine the extent to which maternal milk feeding after very preterm birth is associated with cognitive, academic, and behavioral outcomes at school age and explore whether associations differ according to gestational age at birth, fetal growth status, and infant sex.

## Methods

### Study Design and Participants

For this cohort study, we performed an observational secondary analysis of data from the Docosahexaenoic Acid for Improvement of Neurodevelopmental Outcome (DINO) randomized clinical trial. Briefly, 657 infants born at less than 33 weeks’ gestation without congenital anomalies were enrolled from January 1, 2001, to December 31, 2005, from 5 Australian perinatal centers. Details of recruitment, enrollment, and 7-year follow-up between 2008 and 2013 have been previously published.^12,13^ For this analysis, from the original cohort of 657, we excluded 20 participants who died before 7 years of age and 51 who were not evaluated at 7 years of age, leaving 586 participants. The sample size for each analysis ranged from 508 (77.3% of the original cohort) to 581 (88.4% of the original cohort) infants, depending on available outcome and covariate data. Institutional review boards of all 5 participating centers approved the original study, and parents provided written informed consent for their infant(s) to participate. This secondary data analysis was approved by the Women’s and Children’s Health Network Human Research Ethics Committee, the DINO Trial Steering Committee, and the Partners Human Research Committee. This study followed the Strengthening the Reporting of Observational Studies in Epidemiology (STROBE) reporting guideline.

### Measures

#### Maternal Milk Dose

Once per week, study staff abstracted from the medical record the volume of maternal milk ingested by the infant and the infant’s body weight on that day. From those data, we calculated the mean volume of maternal milk normalized to body weight (milliliters per kilogram daily) fed to each infant during the entire hospitalization, including neonatal intensive care unit (NICU) and special care unit, as described previously.^14^ Pasteurized donor human milk was not used at any site during the study period.

#### Maternal Milk Duration

At follow-up visits at term equivalent age and at 4, 12, and 18 months of corrected age, mothers were asked about the infant’s main source of milk (maternal milk only, both maternal milk and formula, or formula only) and the extent of maternal milk feeding (exclusively maternal milk, maternal milk with 1 or more bottles of formula per day, or exclusively formula fed). If the mother had stopped breastfeeding since the last visit, she was asked on which date she had stopped. To calculate the total duration of maternal milk feeding, including both direct breastfeeding and maternal milk fed via tube and/or bottle, we combined the duration of maternal milk intake while the infant was in the hospital with the duration reported at follow-up visits, as described previously.^14^

### Neurodevelopmental Outcomes

Neurodevelopmental outcomes at 7 years’ corrected age for the DINO trial have been previously reported.^13^ The primary outcome was general intellectual ability, as assessed by the full-scale IQ of the Wechsler Abbreviated Scale of Intelligence (WASI), administered by trained research personnel.^15^ For this secondary analysis, in addition to the full-scale IQ and verbal and performance IQ subscales, we were also interested in outcomes related to academic achievement and behavior, because these are domains in which preterm children perform more poorly than full-term children.^16,17,18^ To measure academic achievement in reading, spelling, and math, trained research personnel administered the Wide Range Achievement Test, Fourth Edition (WRAT-4).^19^ Parents reported symptoms of attention-deficit/hyperactivity disorder (ADHD) with the Conners Third Edition ADHD Index,^20^ behavioral problems with the Strengths and Difficulties Questionnaire (SDQ),^21,22^ and executive function with the Behavioral Rating Inventory of Executive Function (BRIEF).^23^ Both WASI and WRAT-4 are age standardized to a mean (SD) score of 100 (15). The Connors Third Edition ADHD Index T score is age standardized to a mean (SD) score of 50 (10); a normal score is less than 60, the elevated range (indicating more concerns than typical) is 60 to 69, and a very elevated score (indicating many more concerns than typical) is greater than or equal to 70. BRIEF is age standardized to a mean (SD) score of 50 (10); a scale score or index greater than 64 is clinically indicative of dysfunction. In the SDQ, a total difficulties score greater than 13 indicates dysfunction. Higher scores on WASI and WRAT-4 indicate better performance, whereas lower scores on the reported scales from the Conners Third Edition ADHD Index, SDQ total difficulties, and BRIEF indicate better outcomes (fewer problems).

Children who were too impaired to be tested were reviewed by a psychologist and assigned the lowest possible scores. Neither professional assessors nor parents were aware of the hypothesis for this secondary analysis. Assessors were unaware of infant feeding history and were also unaware of the child’s gestational age at birth, although they were aware that the trial enrolled children born at less than 33 weeks’ gestation and in theory could have calculated an individual child’s gestational age at birth from the child’s date of birth and estimated due date or corrected age. Parents could not feasibly be masked to infant feeding or gestational age at birth.

### Covariates

On enrollment, mothers were interviewed about social factors, including income, educational level, occupation, race, smoking status, alcohol use, and the number of people living in the home. Study staff extracted data from the medical record regarding maternal parity and antenatal steroid exposure and infant sex, birth weight, gestational age, multiple birth status, and diagnoses, including necrotizing enterocolitis, bronchopulmonary dysplasia (defined as supplemental oxygen at 36 weeks’ postmenstrual age), sepsis, and severe intraventricular hemorrhage (grade 3 or 4) or periventricular leukomalacia. We derived birth weight for gestational age *z* scores and percentiles using the Fenton reference^24^ and classified infants as small for gestational age (SGA) if the birth weight was less than the 10th percentile.

### Statistical Analysis

Analyses followed a prespecified statistical analysis plan. The exposure variables were mean maternal milk intake volume and duration of maternal milk intake up to a corrected age of 18 months. The main outcome variables were WASI full-scale, verbal, and performance IQ scores; WRAT-4 reading standard, spelling, and math computation scores; ADHD symptoms (Conners Third Edition ADHD Index T score); BRIEF global executive composite score; and SDQ total difficulties score. To estimate the association of the human milk intake with neurodevelopmental outcomes at 7 years of age, we fitted linear models using generalized estimating equations with an independence working correlation structure to account for nonindependence of multiple births and repeated pregnancies and adjusting for clinical and social variables that we conceptualized as potential confounders.^25,26^ Confounders adjusted for in the models included maternal educational level, maternal occupation, number of adults and children living in the home, maternal smoking, maternal alcohol use, parity, race, birth weight (continuous), gestational age (continuous), plurality, exposure to antenatal steroids, treatment group (high vs standard docosahexaenoic acid), and center. We conceptualized maternal race as a social construct. We did not adjust for infant diagnoses because they occurred after the start of measurement of the exposure variables and therefore may be on the causal pathway rather than confounders. We assessed effect modification of the exposure variables (linear continuous) by gestational age (linear continuous), fetal growth status (SGA vs not SGA), sex (female vs male), and treatment group (high docosahexaenoic acid vs control). When looking at effect modification by SGA, we removed birth weight and gestational age as confounders because SGA is defined as birth weight less than the 10th percentile for gestational age. Regarding effect modification for gestational age, we do not report estimates for gestational age categories with fewer than 5 participants. A 2-sided *P* < .05 was considered to be statistically significant. We used R software, version 4.0.3 (R Foundation for Statistical Computing) for all analyses.^27^ The statistical analysis was completed on January 19, 2022.

## Results

From the DINO trial’s original cohort of 657 infants, we excluded 20 participants who died before 7 years of age and 51 participants who were not evaluated at 7 years of age, leaving 586 participants. The 20 infants who died were born at a median gestational age of 25.4 weeks (IQR, 24.9-27.7 weeks). Of survivors, gestational age at birth was similar between participants who were vs were not evaluated at 7 years (median, 30.0 [IQR, 28.0-31.4] vs 30.5 [IQR, 29.5-31.2] weeks). Mothers of participants who were evaluated were more likely to have a tertiary education (245 [51.0%] vs 12 [31.0%]) than mothers of participants who did not participate.

Characteristics of the 586 infants and 486 mothers included in the present cohort are shown in Table 1. Infants were born at a mean (SD) birth weight of 1323 (412) g and gestational age of 29.6 (2.3) weeks; 314 (53.6%) were male and 272 (46.4%) were female. A total of 387 (66.0%) were from singleton gestations. The 486 mothers had a mean (SD) age of 30.4 (5.7) years; 16 (3.3%) were Aboriginal, 17 (3.5%) were Asian, 447 (92.0%) were White, and 6 (1.2%) were other another race; 245 (50.4%) had at least a tertiary education; and 428 (88.0%) had received antenatal steroids. Table 2 lists the exposure and outcome variables. Infants’ mean (SD) maternal milk intake in the NICU was 99 (48) mL/kg daily, and the mean (SD) full-scale IQ score at 7 years of age was 98.5 (13.3) points. At NICU discharge, 541 infants (92.3%) had any maternal milk and 45 (7.7%) did not.

**Table 1.** Infant and Maternal Characteristics^a^

Characteristic	Finding
**Infants (n = 586)**	
Birth weight, mean (SD), g	1312 (412)
Gestational age, mean (SD), wk	29.6 (2.3)
Birth weight for gestational age z score, mean (SD)	−0.03 (0.9)
Sex	
Male	314 (53.6)
Female	272 (46.4)
Singleton	387 (66.0)
Diagnoses	
Chronic lung disease	127 (21.7)
Necrotizing enterocolitis	12 (2.0)
Sepsis	89 (15.1)
IVH grade 3 or 4 or PVL	20 (3.4)
Length of hospital stay, median (IQR), d	
NICU	9 (3-32)
Total hospital stay (NICU and special care nursery)	57 (42-80)
**Mothers (n = 486)**	
Age, mean (SD), y	30.6 (5.5)
Parity	
1	222 (45.6)
2	135 (27.8)
≥3	129 (26.5)
Any smoking in pregnancy	124 (25.5)
Any alcohol use in pregnancy	137 (28.2)
Occupation	
Professional or managerial	109 (22.4)
Semi-skilled, trade, or unskilled	248 (51.0)
Home duties	107 (22.0)
Other	22 (4.5)
Tertiary education	245 (50.4)
Antenatal steroids	428 (88.0)
Race	
Aboriginal	16 (3.3)
Asian	17 (3.5)
White	447 (92.0)
Other^b^	6 (1.2)

Abbreviations: IVH, intraventricular hemorrhage; NICU, neonatal intensive care unit; PVL, periventricular leukomalacia.

^a^ The number of mothers is lower than the number of infants because of multiple gestations and repeated births (3 mothers had 2 births and 1 mother had 3 births). Data are presented as number (percentage) unless otherwise indicated. Percentages may not total to 100% because of missing data (<1% across variables).

^b^ Racial groups included in “Other” are not available.

**Table 2.** Maternal Milk Intake and Duration and Neurodevelopmental Outcomes

Variable	No.	Mean (SD)	Median (IQR)
Maternal milk intake			
Daily volume of maternal milk in the NICU, mL/kg	586	99 (48)	112 (72-133)
Total duration of maternal milk intake, mo	525	5.1 (5.4)	3.1 (1.5-6.7)
Duration of maternal milk feeding after NICU discharge, mo	541	3.1 (4.7)	1.2 (0.1-4.6)
Neurodevelopmental outcomes^a^			
Intelligence (WASI scores)			
Full scale	582	98 (13)	98 (89-107)
Verbal	579	98 (14)	99 (89-108)
Performance	583	99 (13)	96 (89-106)
Academic achievement (WRAT-4 scores)			
Reading standard	575	102 (17)	103 (93-114)
Spelling	570	98 (16)	99 (87-109)
Math computation	576	91 (15)	91 (83-100)
Behavior and executive functioning scores			
ADHD symptoms (Conners Third Edition ADHD Index T score)	575	65 (19)	61 (45-88)
Global executive composite (BRIEF)	574	54.2 (12.7)	53.0 (45.0-62.0)
Total difficulties (SDQ)	577	10.5 (6.5)	10.0 (6.0-14.0)

Abbreviations: ADHD, attention-deficit/hyperactivity disorder; BRIEF, Behavior Rating Inventory of Executive Function; NICU, neonatal intensive care unit; SDQ, Strengths and Difficulties Questionnaire; WASI, Weschler Abbreviated Scale of Intelligence; WRAT-4, Wide Range Achievement Test, Fourth Edition.

^a^ Higher scores on the WASI and WRAT-4 indicate more favorable outcomes, whereas lower score on the Conners Third Edition ADHD Index T score, BRIEF, and SDQ total difficulties indicate more favorable outcomes.

Table 3 shows the unadjusted and adjusted estimates of the difference in neurodevelopmental outcomes at 7 years of age for a unit increase in maternal milk intake. Regarding IQ, a higher intake of maternal milk in the NICU and a longer duration of maternal milk feeding were associated with higher full-scale, verbal, and performance IQ scores in unadjusted models, with 95% CIs that excluded a null effect. Adjustment for clinical and social variables attenuated most of these associations, although maternal milk intake remained positively associated with performance IQ (0.67 points per 25 mL/kg daily; 95% CI, 0.10-1.23 points). Similarly, a higher intake of maternal milk in the NICU and a longer duration of maternal milk feeding were associated with higher reading standard, spelling, and math computation scores in unadjusted models. With adjustment for confounders, these estimates of academic achievement were attenuated, but both maternal milk intake in the NICU and total duration of maternal milk intake remained associated with higher reading standard and math computation scores, and longer duration of milk feeding also remained associated with spelling. For example, for each additional intake of 25 mL/kg daily of maternal milk in the NICU, the reading standard score was 1.14 points higher (95% CI, 0.39-1.89 points), and the math computation score was 0.76 points higher (95% CI, 0.14-1.37 points). With adjustment, longer duration of maternal milk feeding was associated with higher reading standard (0.33 points per month; 95% CI, 0.03-0.63 points per month), math computation (0.30 points per month; 95% CI, 0.03-0.58 points per month), and spelling (0.31 points per month; 95% CI, 0.01-0.62 points per month) scores. Higher maternal milk intake in the NICU was also associated with a lower (more favorable) Conners Third Edition ADHD Index T score (1.08 lower per 25 mL/kg daily of maternal milk; 95% CI, −1.96 to −0.20 in the adjusted model) but not with BRIEF or SDQ scores.

**Table 3.** Associations of Maternal Milk Intake With Neurodevelopmental Outcomes at School Age^a^

Model	β (95% CI)		
**IQ (WASI** ^b^ **)**			
Outcome	Full scale (n = 580)	Verbal (n = 577)	Performance (n = 581)
Per 25 mL/kg daily in the NICU			
Crude	1.01 (0.42 to 1.60)	0.69 (0.05 to 1.33)	1.04 (0.47 to 1.60)
Adjusted	0.40 (−0.16 to 0.96)	−0.03 (−0.64 to 0.58)	0.67 (0.10 to 1.23)
Per month			
Crude	0.42 (0.17 to 0.66)	0.35 (0.10 to 0.60)	0.39 (0.14 to 0.63)
Adjusted	0.22 (−0.03 to 0.471	0.18 (−0.07 to 0.43)	0.22 (−0.03 to 0.46)
**Academic achievement (WRAT-4** ^b^ **)**			
Outcome	Reading standard (n = 573)	Spelling (n = 568)	Math computation (n = 574)
Per 25 mL/kg daily in the NICU			
Crude	1.64 (0.90 to 2.37)	1.01 (0.33 to 1.70)	1.26 (0.62 to 1.90)
Adjusted	1.14 (0.39 to 1.89)	0.59 (−0.11 to 1.29)	0.76 (0.14 to 1.37)
Per month			
Crude	0.48 (0.19 to 0.78)	0.42 (0.13 to 0.72)	0.44 (0.17 to 0.70)
Adjusted	0.33 (0.03 to 0.63)	0.31 (0.01 to 0.62)	0.30 (0.03 to 0.58)
**Behavior and executive functioning**			
Outcome	ADHD symptoms to Conners Third Edition ADHD Index T score (n = 573)	Global executive composite to BRIEF (n = 572)	Total difficulties to SDQ (n = 575)
Per 25 mL/kg daily in the NICU			
Crude	−0.96 (−1.81 to −0.10)	−0.45 (−1.09 to 0.19)	−0.26 (−0.58 to 0.06)
Adjusted	−1.08 (−1.96 to −0.20)	−0.45 (−1.10 to 0.20)	−0.22 (−0.54 to 0.10)
Per month^c^			
Crude	−0.20 (−0.51 to 0.10)	−0.08 (−0.28 to 0.12)	−0.06 (−0.17 to 0.05)
Adjusted	−0.13 (−0.44 to 0.19)	−0.04 (−0.24 to 0.17)	−0.01 (−0.13 to 0.10)

Abbreviations: ADHD, attention-deficit/hyperactivity disorder; BRIEF, Behavior Rating Inventory of Executive Function; NICU, neonatal intensive care unit; SDQ, Strengths and Difficulties Questionnaire; WASI, Weschler Abbreviated Scale of Intelligence; WRAT-4, Wide Range Achievement Test, Fourth Edition.

^a^ The crude model is unadjusted. The adjusted model is adjusted for treatment, center, maternal tertiary education, maternal occupation, number of adults and children in the home, maternal smoking and alcohol use in pregnancy, parity, race, birth weight (continuous), gestational age (continuous), and antenatal steroid exposure. Generalized estimating equations were used to account for clustering for multiple gestation.

^b^ Higher scores on WASI and WRAT-4 indicate more favorable outcomes, whereas lower score on the Conners Third Edition ADHD Index, BRIEF, and SDQ total difficulties scales indicate more favorable outcomes.

^c^ Numbers are smaller because of missing data on duration of human milk intake after NICU discharge.

Table 4 gives the adjusted estimates of the difference in neurodevelopmental outcomes at 7 years of age for a higher maternal milk intake of 25 mL/kg daily in the NICU, modified by gestational age. The positive association between maternal milk intake and IQ and academic achievement scores was stronger among infants born at the lowest gestational ages, with effects generally confined to infants born at less than 30 weeks of gestation. The exception was for Conners Third Edition ADHD Index T scores for ADHD, where the beneficial associations were strongest among infants born at greater than 29 weeks of age (*P* for interaction = .04). Regarding SGA status (Table 5), maternal milk intake was associated with better performance IQ for non-SGA infants only (*P* for interaction = .03). There was no evidence of effect modification by infant sex (eTable in the Supplement) or original DINO treatment group. To facilitate comparison with a previous publication,^28^ we conducted a post hoc analysis of the SDQ subscales and found no association between duration of maternal milk intake and conduct or inattention/hyperactivity scores.

**Table 4.** Associations of Maternal Milk Intake in the NICU With Neurodevelopmental Outcomes at School Age: Modification by Gestational Age^a^

Gestational age at birth, wk	No.	β (95% CI)	*P* value for interaction	β (95% CI)	*P* value for interaction	β (95% CI)	*P* value for interaction
**Intelligence (WASI** ^b^ **)**							
Outcome		Full scale	<.001	Verbal	.004	Performance	.004
25	32	2.17 (1.00 to 3.34)		1.63 (0.33 to 2.93)		2.12 (1.04 to 3.20)	
26	26	1.78 (0.81 to 2.76)		1.27 (0.18 to 2.36)		1.81 (0.90 to 2.71)	
27	30	1.40 (0.60 to 2.20)		0.91 (0.02 to 1.80)		1.49 (0.75 to 2.24))	
28	50	1.02 (0.37 to 1.66)		0.54 (−0.18 to 1.27)		1.18 (0.56 to 1.80	
29	54	0.63 (0.08 to 1.19)		0.18 (−0.44 to 0.80)		0.86 (0.31 to 1.42)	
30	79	0.25 (−0.30 to 0.80)		−0.18 (−0.78 0.41)		0.55 (−0.02 to 1.11)	
31	100	−0.14 (−0.77 to 0.49)		−0.54 (−1.21 to 0.12)		0.23 (−0.41 to 0.88	
32	96	−0.52 (−1.29 to 0.25)		−0.91 (−1.72 to −0.10)		−0.08 (−0.86 to 0.70)	
33	107	−0.91 (−1.85 to 0.04)		−1.27 (−2.26 to −0.28)		−0.40 (−1.34 to 0.55)	
**Academic achievement (WRAT-4** ^b^ **)**							
Outcome		Reading standard	<.001	Spelling	<.001	Math computation	.02
25	32	3.48 (1.84 to 5.13)		2.81 (1.31 to 4.31)		2.02 (0.76 to 3.29)	
26	25	2.98 (1.60 to 4.37		2.33 (1.07 to 3.60)		1.75 (0.68 to 2.82)	
27	29	2.49 (1.34 to 3.63)		1.86 (0.81 to 2.91)		1.48 (0.60 to 2.37)	
28	50	1.99 (1.06 to 2.91)		1.39 (0.52 to 2.25)		1.21 (0.48 to 1.95)	
29	51	2.49 (0.71 to 2.26)		0.91 (0.18 to 1.64)		0.94 (0.31 to 1.58)	
30	78	0.99 (0.27 to 1.70)		0.44 (−0.24 to 1.12)		0.67 (0.06 to 1.29)	
31	100	0.49 (−0.29 to 1.26)		−0.03 (−0.77 to 0.71)		0.41 (−0.27 to 1.08)	
32	96	−0.01 (−0.94 to 0.92)		−0.51 (−1.38 to 0.37)		0.14 (−0.67 to 0.94)	
33	107	−0.51 (−1.66 to 0.63)		−0.98 (−2.04 to 0.09)		−0.13 (−1.11 to 0.84)	
**Behavior and executive functioning**							
Outcome		ADHD symptoms to Conners Third Edition ADHD Index T score	.04	BRIEF global executive composite	.39	SDQ total difficulties	.22
25	32	0.59 (−1.29 to 2.47)		0.06 (−1.27 to 1.39)		0.12 (−0.51 to 0.75)	
26	26	0.22 (−1.36 to 1.81		−0.05 (−1.17 to 1.07)		0.05 (−0.48 to 0.58)	
27	31	−0.14 (−1.45 to 1.17)		−0.16 (−1.09 to 0.77)		−0.02 (−0.47 to 0.42)	
28	51	−0.50 (−1.58 to 0.57)		−0.26 (−1.04 to 0.51)		−0.10 (−0.47 to 0.27)	
29	54	−0.87 (−1.78 to 0.05)		−0.37 (−1.04 to 0.30)		−0.17 (−0.50 to 0.15)	
30	79	−1.23 (−2.09 to −0.37)		−0.48 (−1.14 to 0.18)		−0.25 (−0.57 to 0.08)	
31	98	−1.59 (−2.53 to −0.66)		−0.59 (−1.32 to 0.14)		−0.32 (−0.68 to 0.04)	
32	92	−1.96 (−3.07 to −0.84)		−0.70 (−1.57 to 0.17)		−0.39 (−0.83 to 0.04)	
33	104	−2.32 (−3.68 to −0.96)		−0.80 (−1.85 to 0.25)		−0.47 (−0.99 to 0.05)	

Abbreviations: ADHD, attention-deficit/hyperactivity disorder; BRIEF, Behavior Rating Inventory of Executive Function; NICU, neonatal intensive care unit; SDQ, Strengths and Difficulties Questionnaire; WASI, Weschler Abbreviated Scale of Intelligence; WRAT-4, Wide Range Achievement Test, Fourth Edition.

^a^ Gestational age at birth was fitted as a continuous variable. The β estimates indicate the difference in points per 25 mL/kg daily of additional maternal milk intake in the NICU for an exact gestational age (eg, 25 weeks plus 0 days). The sample size provided for each gestational age is the number of participants in the gestational age category (eg, 25 completed weeks [25 weeks plus 0 days through 25 weeks plus 6 days]). Estimates for gestational age categories with fewer than 5 participants are not reported. *P* values are for the interaction of gestational age interval. All estimates are adjusted for treatment, center, maternal tertiary education, maternal occupation, number of adults and children in the home, maternal smoking and alcohol use in pregnancy, parity, race, birth weight (continuous) and antenatal steroid exposure. Generalized estimating equations were used to account for clustering for multiple gestation. Sample size may be slightly different across subscales.

^b^ Higher scores on WASI and WRAT-4 indicate more favorable outcomes, whereas lower score on the Conners Third Edition ADHD Index, BRIEF, and SDQ total difficulties scales indicate more favorable outcomes.

**Table 5.** Associations of Maternal Milk Intake in the NICU With Neurodevelopmental Outcomes at School Age: Modification by Fetal Growth Status^a^

Fetal growth status	No.	β (95% CI)	*P* value for interaction	β (95% CI)	*P* value for interaction	β (95% CI)	*P* value for interaction
**Intelligence (WASI** ^b^ **)**								
Outcome		Full scale	.07	Verbal	.28	Performance	.03
SGA	49	−1.52 (−3.65 to 0.61)		−1.49 (−4.20 to 1.23)		−1.40 (−3.35 to 0.55)	
Not SGA	532	0.52 (−0.09 to 1.13)		0.04 (−0.62 to 0.71)		0.81 (0.22 to 1.39)	
**Academic achievement (WRAT-4** ^b^ **)**								
Outcome		Reading standard	.79	Spelling	.19	Math computation	.24
SGA	49	0.73 (−2.34 to 3.81)		−1.12 (−3.75 to 1.51)		−0.70 (−3.22 to 1.81)	
Not SGA	525	1.15 (0.36 to 1.94)		0.67 (−0.08 to 1.42)		0.82 (0.17 to 1.48)	
**Behavior and executive functioning**								
Outcome		ADHD symptoms to Conners T score	.77	Global executive composite to BRIEF	.11	Total difficulties to SDQ	.38
SGA	49	−0.65 (−3.65 to 2.34)		0.99 (−0.84 to 2.82)		0.24 (−0.84 to 1.31)	
Not SGA	526	−1.12 (−2.03 to −0.21)		−0.52 (−1.20 to 0.16)		−0.25 (−0.59 to 0.08)	

Abbreviations: ADHD, attention-deficit/hyperactivity disorder; BRIEF, Behavior Rating Inventory of Executive Function; NICU, neonatal intensive care unit; SDQ, Strengths and Difficulties Questionnaire; SGA, small for gestational age (birth weight <10th percentile); WASI, Weschler Abbreviated Scale of Intelligence; WRAT-4, Wide Range Achievement Test, Fourth Edition.

^a^ The β estimates indicate points per 25 mL/kg daily of additional maternal milk intake in the NICU. *P* values are for the interaction of SGA category. All estimates are adjusted for treatment, center, maternal tertiary education, maternal occupation, number of adults and children in the home, maternal smoking in pregnancy, parity, race, and antenatal steroid exposure. Generalized estimating equations were used to account for clustering for multiple gestation. Sample size may be slightly different across subscales.

^b^ Higher scores on the WASI and WRAT-4 indicate more favorable outcomes, whereas lower score on the Conners Third Edition ADHD Index, BRIEF, and SDQ total difficulties indicate more favorable outcomes.

## Discussion

In this large, longitudinal cohort study of preterm infants born at less than 33 weeks’ gestation, we found that higher maternal milk intake during the NICU hospitalization was associated with higher performance IQ, better academic achievement in reading and math, and fewer parent-reported symptoms of ADHD at school age, even after adjustment for clinical and social confounders. These associations are clinically important in magnitude. For example, 1 point on the reading scale per each additional 25 mL/kg daily of maternal milk translates to approximately 6 to 7 points (more than one-third of an SD) when multiplied over the typical daily intake in the NICU, which is 160 to 180 mL/kg daily. In addition, we found that the beneficial associations of maternal milk feeding with neurodevelopment were stronger for infants born at the lowest gestational ages.

Our findings substantially extend previous literature on the neurodevelopmental benefits of maternal milk feeding for preterm infants. Of relevance to current practice are studies conducted during the era of routine human milk fortification. Most of those studies report outcomes in infants and toddlers^6,7^ and are therefore limited given the substantial evolution in cognitive performance that occurs in very preterm–born children over time,^29^ as well as the relatively small range of cognitive and behavioral domains that are testable in young children. A few previous studies have reported school-age outcomes, but most lack detail on the dose and/or timing of maternal milk feeding, an important limitation given the typical decrease in maternal milk provision after the first month in the NICU^30^ and given evidence that the NICU hospitalization is a particularly sensitive period for preterm brain development related to nutritional exposures.^31^

Consistent with our study, in a large, regional French cohort born in 1997, maternal milk feeding, compared with formula feeding, assessed at the time of NICU discharge, was associated with a protective effect against low IQ at age 5 years.^32^ A US study^33^ of more than 400 infants with very low birth weight (<1500 g) found that parent-reported feeding (direct breastfeeding vs no direct or expressed milk feeding) was associated with better visual motor integration at age 6 to 8 years, but associations with other measures were attenuated with full adjustment for confounders. A previous study^8^ in a different Australian cohort quantified the number of days on which maternal milk comprised greater than 50% of enteral intake during the first month of life and found positive associations with IQ, mathematics, working memory, and motor function tests at 7 years of age. We also found that a longer duration of maternal milk feeding, including the period after hospital discharge, was associated with benefits for academic achievement in terms of reading, spelling, and math scores, although the effect sizes were more modest than for the intake of maternal milk during the hospitalization. These findings are consistent with previously reported literature.^32^ Taken together, the available evidence supports the concept that a higher dose of maternal milk during the NICU hospitalization, and possibly after discharge, is associated with beneficial effects at school age on intelligence and academic achievement, particularly in mathematics given the consistency in this association across 2 separate cohorts.

In addition to associations with IQ and academic achievement, we noted that a higher dose of maternal milk was associated with lower Conners Third Edition ADHD Index T scores, indicating fewer symptoms of ADHD as reported by parents. Very preterm infants are at heightened risk of ADHD relative to their peers born at full term.^34^ Data linking maternal milk or breastfeeding with symptoms of ADHD in the preterm population are sparse, but our findings are consistent with a German Neonatal Network study^35^ in which parent-reported breastfeeding for 3 months or longer was associated with lower conduct and inattention/hyperactivity scores, indicating fewer problems in these domains, as assessed by the SDQ. Our finding of no association between duration of maternal milk intake and the SDQ inattention/hyperactivity scores may reflect the poorer sensitivity of the SDQ in detecting ADHD symptoms compared with the Conners Third Edition ADHD Index.^36^ Several studies in full-term populations have linked breastfeeding duration with ADHD diagnosis and/or related symptoms,^37,38,39^ whereas other studies, including a large randomized clinical trial of breastfeeding promotion,^40^ have reported no association.^41^ Notably, generalizability from studies of full-term infants to very preterm infants is limited given differences in timing of exposure with respect to underlying brain developmental processes^42^ and given the increased underlying risk of behavioral difficulties in preterm-born children.^43^ Overall, cumulative evidence to date suggests a possible protective effect of maternal milk against ADHD symptoms at school age in very preterm-born children, but firm conclusions are limited because of a lack of data from very preterm cohorts.

The findings regarding ADHD symptoms may also help to explain another observation—that associations of maternal milk were stronger and more consistent with academic achievement than with IQ. It is plausible that an underlying attentional vulnerability may manifest as impaired school performance, independent of IQ, as has been observed in both general and very preterm cohorts.^44,45^ Typically, attention problems also impact cognitive testing but often less markedly than school performance.^46^

A beneficial association of maternal milk with school-age neurodevelopmental outcomes is biologically plausible when framed within the Developmental Origins of Health and Disease paradigm and in the context of mounting research describing the myriad, potentially beneficial nutrient and nonnutrient bioactive factors in human milk.^47^ Maternal milk feeding during NICU hospitalization has been linked in several studies^8,9,10,11^ with differences in brain structure on magnetic resonance imaging at term-equivalent age, suggesting short-term effects during a critical period in development. Insights from animal models and human studies that link variation in exposure to specific milk nutrients and/or nonnutrient bioactive factors, or mixtures of these nutrients and bioactive factors, with early brain development and neurodevelopmental outcomes can inform randomized clinical trials and eventually new clinical strategies for supplementation.^48^

### Strengths and Limitations

This study has several strengths, including the large sample size, which allowed us to adjust simultaneously for multiple covariates and to perform analyses stratified by gestational age. High retention of the cohort through 7 years of age further strengthens internal validity. The study also has limitations. The main findings were robust to adjustment for potential medical and social confounders, but as with all observational studies, this study is limited by the potential for residual confounding by unmeasured factors in the NICU or home environment. The use of developmental therapies may have differed by feeding status, but these differences would be on the causal pathway and therefore should not be adjusted for. Given the large number of outcomes, some statistically significant findings may be spurious, although all were in the hypothesized direction, and 95% CIs provide information about the precision of estimates. Generalizability to very preterm infant populations outside Australia, particularly infants born in low-resource settings, may be limited, and racial and ethnic diversity of the cohort is also lacking.

## Conclusions

In this cohort study, we found that a higher dose of maternal milk during the critical period from very preterm birth to term-equivalent age was associated with improved cognitive and academic outcomes and fewer ADHD symptoms at school age. These associations were stronger for infants born before 30 weeks, suggesting that infants who are less mature at birth may derive greater benefit from maternal milk feeding after birth. These findings affirm recommendations by national and international organizations that fortified maternal milk should be the primary diet for infants born very preterm.^44,45,46^ In addition, these findings suggest that hospital-based and societal efforts to increase maternal milk feeding through policies that protect, promote, and support lactation for women who deliver very preterm are well justified based on long-term benefits to child neurodevelopment.

## Notes

Supplement. **eTable.** Associations of Maternal Milk Intake in NICU With Neurodevelopmental Outcomes at School Age: Modification by Sex
