# Supplementary material for: Associations of Maternal Milk Feeding With Neurodevelopmental Outcomes at 7 Years of Age in Former Preterm Infants
Source: JAMA Netw Open. 2022 Jul 13;5(7):e2221608. doi: 10.1001/jamanetworkopen.2022.21608 (PMC9280396; doi:10.1001/jamanetworkopen.2022.21608)
Supplement: Supplement. — eTable. Associations of Maternal Milk Intake in NICU With Neurodevelopmental Outcomes at School Age: Modification by Sex [file jamanetwopen-e2221608-s001.pdf]

Supplementary Online Content

Belfort MB, Knight E, Chandarana S, et al. Associations of maternal milk feeding with neurodevelopmental outcomes at 7 years of age in former preterm infants. *JAMA Netw Open*. 2022;5(7):e2221608. doi:10.1001/jamanetworkopen.2022.21608

eTable. Associations of Maternal Milk Intake in NICU With Neurodevelopmental Outcomes at School Age: Modification by Sex

This supplementary material has been provided by the authors to give readers additional information about their work.

© 2022 Belfort MB et al. *JAMA Network Open.*

**eTable.** Associations of Maternal Milk Intake in NICU With Neurodevelopmental Outcomes at School Age: Modification by Sex										
		Intelligence (WASI)								
		Full Scale			Verbal			Performance		
Infant sex	N	Beta	95% CI	$P_{interaction}$	Beta	95% CI	$P_{interaction}$	Beta	95% CI	$P_{interaction}$
Female	270	0.53	-0.26, 1.32	0.74	0.18	-0.70, 1.05	0.58	0.73	-0.04, 1.50	0.89
Male	310	0.35	-0.38, 1.08		-0.16	-0.98, 0.66		0.66	-0.05, 1.38	
		Academic achievement (WRAT)								
		Reading			Spelling			Math		
		Beta	95% CI	$P_{interaction}$	Beta	95% CI	$P_{interaction}$	Beta	95% CI	$P_{interaction}$
Female	268	1.38	0.34, 2.42	0.59	1.05	0.09, 2.02	0.20	0.55	-0.26, 1.36	0.42
Male	305	0.99	-0.02, 2.00		0.19	-0.74, 1.13		1.00	0.17, 1.82	
		Behavior and executive functioning								
		ADHD symptoms, Conners T score			Global executive composite, BRIEF			Total difficulties, SDQ		
		Beta	95% CI	$P_{interaction}$	Beta	95% CI	$P_{interaction}$	Beta	95% CI	$P_{interaction}$
Female		-0.99	-2.30, 0.31	0.82	-0.33	-1.29, 0.64	0.70	-0.29	-0.75, 0.16	0.66
Male		-1.18	-2.27, -0.09		-0.56	-1.35, 0.23		-0.16	-0.57, 0.25	

Beta estimates indicate points per 25 mL/kg/day additional maternal milk intake in the neonatal intensive care unit (NICU). P-values are for the interaction of sex category. WASI is Weschler Abbreviated Scale of Intelligence. WRAT is Wide Range Achievement Test. ADHD is attention deficit hyperactivity disorder. BRIEF is Behavior Rating Inventory of Executive Function. SDQ is Strengths and Difficulties Questionnaire. Higher scores on the WASI and WRAT indicate more favorable outcomes, whereas lower score on the Conners, BRIEF, and SDQ total difficulties indicate more favorable outcomes. All estimates are adjusted for treatment, center, maternal tertiary education, maternal occupation, number of adults and children in the home, maternal smoking and alcohol use in pregnancy, parity, race and antenatal steroid exposure. Generalized estimating equations were used to account for clustering due to multiple gestation. Sample size may be slightly different across subscales.

© 2022 Belfort MB et al. *JAMA Network Open.*
